# Comparative Effectiveness of Daily Supportive Text Messages Versus Email Messages for Patients with Depression. Randomized Hybrid Type II Effectiveness-Implementation Trial

**DOI:** 10.1192/j.eurpsy.2023.1775

**Published:** 2023-07-19

**Authors:** M. K. Adu, R. Shalaby, E. Eboreime, V. Agyapong

**Affiliations:** 1 Psychiatry, Dalhousie University, Halifax NS, Halifax; 2 Psychiatry, University of Alberta, Edmonton, Canada

## Abstract

**Introduction: Background:**

Major depressive disorder (MDD) is a global health problem accounting for about 40.5% of disability-adjusted life years caused by mental and substance use disorders. Barriers to accessing healthcare services have been reported, highlighting the need for innovative, accessible, and cost-effective psychological interventions. Several clinical trials have proven the effectiveness of supportive SMS text messaging in ameliorating depressive symptoms, however, this approach can only be accessible to individuals having cell phones.

**Objectives:**

This paper aims to evaluate the effectiveness, feasibility, and user satisfaction of daily supportive email messaging as a non-inferior intervention compared to daily supportive text messaging as an add-on treatment for patients with depression.

**Methods:**

This trial will be carried out using a hybrid type II implementation-effectiveness design. In addition to the usual care, patients with depression will be randomized to receive either supportive text messages or supportive email messages. The messages in both groups will have the same content and will be provided daily for 6 months. The implementation evaluation will be guided by the Consolidated Framework for Implementation Research and the RE-AIM (Reach, Effectiveness, Adoption, Implementation, and Maintenance) framework. Descriptive and inferential statistics will be employed in the analysis of the quantitative outcome measures, while thematic analysis will be used for Qualitative data.

**Results:**

The results are expected to be available 18 months after the start of recruitment. The results will highlight the feasibility, acceptability, and effectiveness of using automated emails as a strategy for delivering supportive messages to patients with depression as non-inferior to text messaging.

**Conclusions:**

The outcome of this trial will have a translational impact on routine patient care and access to mental health, as well as potentially support mental health policy decision-making for health care resource allocation.

**Disclosure of Interest:**

None Declared

